# Applications of Extreme Value Theory in Public Health

**DOI:** 10.1371/journal.pone.0159312

**Published:** 2016-07-15

**Authors:** Maud Thomas, Magali Lemaitre, Mark L. Wilson, Cécile Viboud, Youri Yordanov, Hans Wackernagel, Fabrice Carrat

**Affiliations:** 1 Department of Mathematical Statistics, Chalmers University of Technology–Göteborg, Sweden; 2 Sorbonne Universités, UPMC Univ Paris 06, INSERM, Institut Pierre Louis d’Epidémiologie et de Santé Publique, UMR_S 1136, F-75012, Paris, France; 3 Fogarty International Center, NIH, Washington, DC, United States of America; 4 Department of Epidemiology, School of Public Health, University of Michigan, Ann Arbor, Michigan, United States of America; 5 Service des Urgences, Hôpital Saint-Antoine, Assistance Publique-Hôpitaux de Paris, Paris, France; 6 Geostatistics group, Centre de Géosciences, MINES ParisTech, PSL Research University, Fontainebleau, France; 7 Public Health Unit, Saint-Antoine hospital, AP-HP, F-75012, Paris, France; University of Waterloo, CANADA

## Abstract

**Objectives:**

We present how Extreme Value Theory (EVT) can be used in public health to predict future extreme events.

**Methods:**

We applied EVT to weekly rates of Pneumonia and Influenza (P&I) deaths over 1979–2011. We further explored the daily number of emergency department visits in a network of 37 hospitals over 2004–2014. Maxima of grouped consecutive observations were fitted to a generalized extreme value distribution. The distribution was used to estimate the probability of extreme values in specified time periods.

**Results:**

An annual P&I death rate of 12 per 100,000 (the highest maximum observed) should be exceeded once over the next 30 years and each year, there should be a 3% risk that the P&I death rate will exceed this value. Over the past 10 years, the observed maximum increase in the daily number of visits from the same weekday between two consecutive weeks was 1133. We estimated at 0.37% the probability of exceeding a daily increase of 1000 on each month.

**Conclusion:**

The EVT method can be applied to various topics in epidemiology thus contributing to public health planning for extreme events.

## Introduction

A central question for resource planning in public health is to predict the likelihood that exceptional or extreme events will occur in the not too distant future [1]. Such events might be, for example, an unusual community epidemic, a major heat wave, or an accidental toxic exposure. Statistical approaches focused on *extreme values* have shown promising results in forecasting unusual events in earth sciences, genetics and finance. For instance, *Extreme Value Theory* (EVT) was developed in the 1920s [2] and has been used to predict the occurrence of events as varied as droughts and flooding [3] or financial crashes [4]. To our knowledge, applications of EVT in public health are scarce. In a first unpublished work we applied the method to predict extreme influenza mortality in the US [5]. EVT was also used to detect outliers (here seen as extreme events) in time series surveillance data, rather than estimate the risk of future extreme events [6]. More recently, a study applied EVT to predict monthly incidence of avian influenza cases [7].

The main goal of EVT is to assess, from a series of observations, the probability of events that are more extreme than those previously recorded. For example, 40% of the Netherlands is below the sea level and has to be protected against the sea by dikes. The height of dikes can be calculated from storm data collected for around 100 years using EVT, so that the risk of flooding would be less than one every 10,000 years [8]. Similarly, the likelihood of epidemics of unusual sizes could be determined by applying EVT to past epidemic observations, which could then help planning resources for mitigating the burden of these epidemics. The aim of this paper is to present how EVT can be applied in public health. We illustrate its use on two different applications–to predict extremes of annual seasonal influenza mortality or variations by weekday in daily number of emergency department visits.

## Materials and Methods

### Ethics statement

Data used in this paper were counts of deaths (per age group and per month) or counts of emergency visits (per day) or counts of population (per age group).

All data were received by the authors in de-identified form. These data were strictly anonymous and did not require approval from an ethics committee.

### Extreme Value Theory (EVT)–Block Maxima Method

Based on EVT [2], the limit distribution of the maximum of *n* random variables belongs to a three-parameter Generalized Extreme Value (GEV) family, and has a cumulative distribution function of the form:

$$G(z)=\left\{ \begin{array}{l} exp\{-{\left[1+\xi (\frac{z-\mu }{\sigma })\right]}^{-1/\xi }\},\;\xi \neq 0\\ \;exp\{-e^{-(z-\mu )/\sigma }\}\;,\;\xi =0 \end{array}\right.$$

where *μ* is a location parameter, *σ* a scale parameter and *ξ* a shape parameter. G is defined for all z such that (1 + *ξ* (*z* − *μ*)/*σ*) > 0 for *ξ* ≠ 0 and all z for *ξ* = 0. Three classes of GEV distributions are defined depending on the value of *ξ*:

- The Fréchet class (*ξ* > 0), representing distributions with heavy tail.
- The Gumbel class (*ξ* = 0), representing distributions with lighter tail.
- The Weibull class (*ξ* < 0), representing distributions with finite tail.

A classical method for modelling the extremes of a stationary time series is the method of block maxima, in which consecutive observations are grouped into non-overlapping blocks of length *n*, generating a series of *m* block maxima, M_n,1_,…, M_n,m_, say, to which the GEV distribution can be fitted for some large value of *n*. The usual approach is to consider blocks of a given time length (e.g. a month, a year), thus yielding maxima at regular intervals (monthly, annual) [2].

Once a GEV distribution is fitted to *n* empirical observations, it becomes possible (1) to estimate the probability of an event that has not been observed yet, *e*.*g*. the probability to exceed a certain threshold larger than the largest observation, and (2) to estimate an extreme quantile, *e*.*g*. a quantile of order of 1-1/(*xn*) where *x* is much larger than 1. This is an extreme quantile because only *n* observations are included in the block. The first estimate is simply given by the distribution function of the GEV. The (1-*p*)-quantile *z*_*p*_ of a GEV distribution is called the return level associated with the return period *t*_*p*_ *(= 1/p)*. For blocks of one year, the level *z*_*p*_ is expected to be exceeded on average once every years *t*_*p*_. More precisely, *z*_*p*_ is exceeded by the annual maximum in any particular year with probability *p*. The level *z*_*p*_ can be expressed in terms of the GEV parameters:

$$z_{p}=\left\{ \begin{array}{l} \mu -\frac{\sigma }{\xi }[1-{\left\{-\log\left(1-p\right)\right\}}^{-\xi }],\;\xi \neq 0\\ \mu -\sigma \;\log\left\{-\log\left(1-p\right)\right\}\;,\;\xi =0 \end{array}\right.$$

We used the *evd* and *extRemes* packages in R v 3.0.2 for GEV calculations[2]. We used Mathematica® v10 (Wolfram Research Inc, Champaign, IL USA) and a built-in Kolmogorov Smirnov (KS) test with critical values determined by 10,000 Monte Carlo simulations to assess the fit of the GEV distribution.

## Results

### Pneumonia and Influenza mortality

Pneumonia and Influenza (P&I) mortality data provide a specific indicator of influenza mortality [9]. We obtained weekly number of P&I deaths in France from July 1979 to June 2011 from death certificates collected by the Centre d’épidémiologie sur les causes médicales de décès. We used the following codes from the International Classification of Diseases (ICD): 480–487 (1979–1999 ICD-9th revision) and J09-J18 (2000–2011, ICD-10th revision). Mortality data were split into 9 age groups (0–4, 5–14, 15–24, 25–34, 35–44, 45–54, 55–64, 65–74, ≥75). Population data obtained from regular censuses were used to calculate weekly age-specific P&I mortality rates. We then calculated weekly age-standardized death rates using the 2011 French population structure as reference.

**Fig 1:**
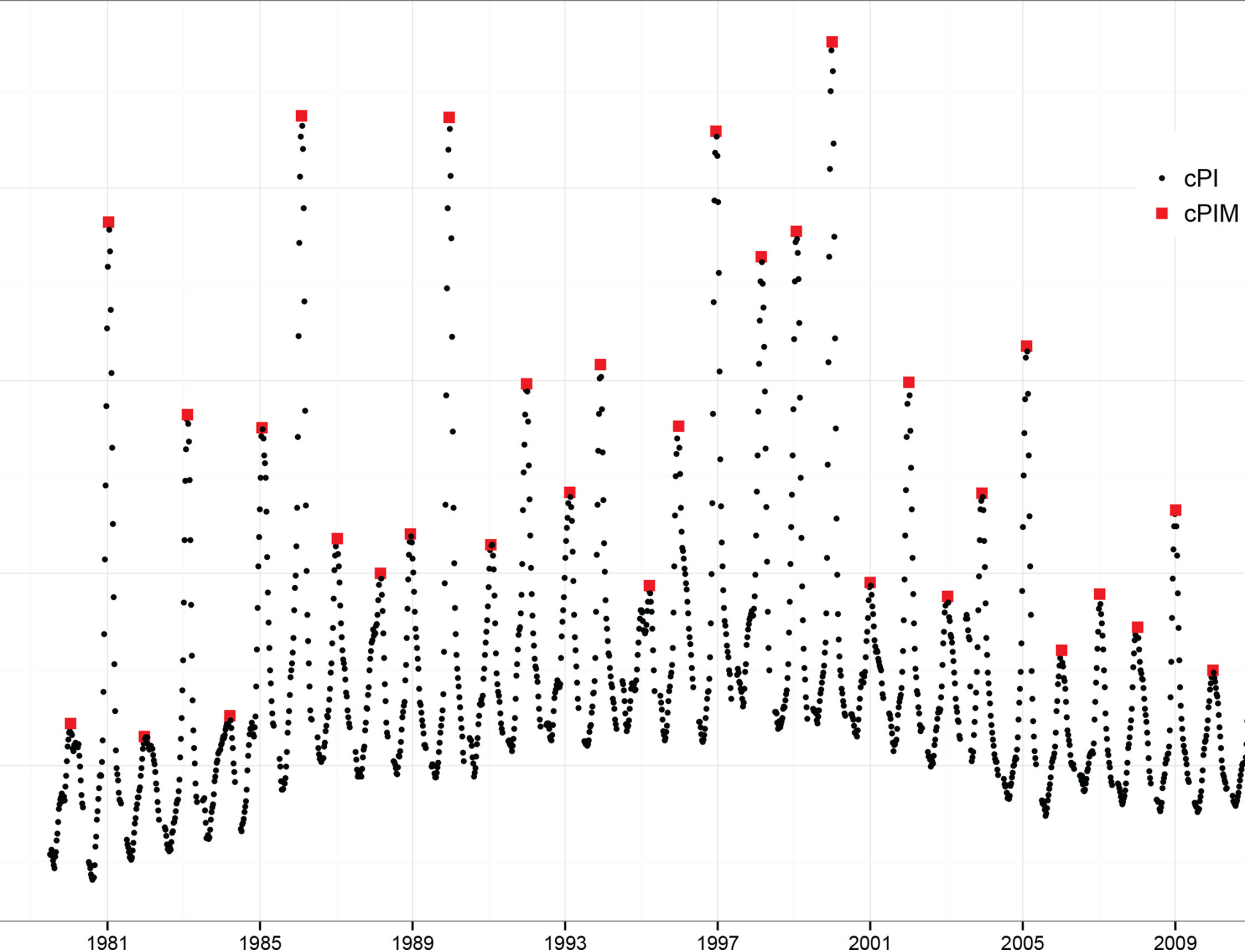
Age-standardized cumulative P&I mortality rates (1979–2011) in France. cPI rates correspond to black symbols and cPIM, the annual maxima, to the red symbols.

We defined the cumulative rates of P&I mortality (cPI) as the sum of weekly P&I mortality over eight consecutive weeks using a moving time window, through the entire time series (Fig 1). The eight-week period coincides with the length of a typical influenza epidemic, which is estimated to last approximately 2 months on average.

In our application, M_n,1_,…, M_n,m_ stand for the maxima of *n* cPI observations within a respiratory year that is, from July to June to encompass annual influenza epidemics. We obtained 32 annual maxima, denoted cPIM (Fig 1). The highest maximum (12 deaths per 100,000) was observed during the 1999–2000 respiratory year.

**Fig 2:**
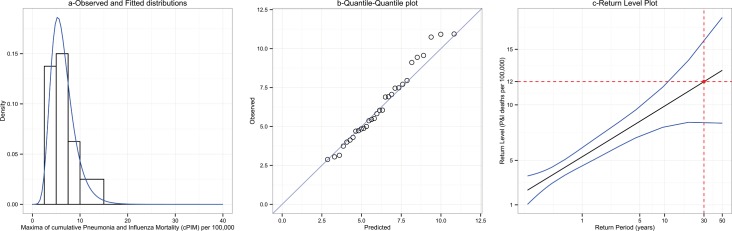
Fit of the Generalized Extreme Value distribution to the annual maxima of cumulative P&I mortality rates. a-Empirical (bars) and fitted (curve) distributions for the annual maxima of cumulative P&I mortality. b-Quantile-Quantile (QQ) plots for the annual maxima of cumulative P&I mortality. c- Return plots for the annual maxima of cumulative P&I mortality

Assuming that cPIM were distributed according to a GEV, we estimated the GEV parameters and their 95% Confidence Intervals (95%CI) by the maximum-likelihood method: the location parameter (*μ*) estimate was 5.33, 95%CI (4.51;6.14), the scale parameter (σ) estimate was 1.97, 95%CI (1.35;2.59), and the shape parameter (*ξ*) estimate was 0.004, 95%CI (-0.36;0.37). The fit of the GEV distribution was correct (KS test P-value = 0.79), as shown by the empirical and fitted cPIM distributions (Fig 2A) and the Quantile-Quantile plot, *i*.*e*. the observed and predicted quantiles agreed overall (Fig 2B).

Return level plots were then calculated for return periods up to 50 years (Fig 2C). Note that the linear aspect of the plot was the consequence of the close-to-zero shape parameter. This plot suggests that over the next 30 years (the return period), the cPIM should exceed 12, 95%CI (8; 16) deaths per 100,000 (the return level) once. Another way to interpret the plot is to say that there is approximately a 3% chance (1/30) each year that the cPIM will exceed 12 deaths per 100,000. Finally, we computed the probabilities to exceed some cPIM values greater than the largest maxima ever observed. As an example, there is a 1.11% chance that the cPIM of a given year will exceed 14 deaths per 100,000, a 0.19% chance that the cPIM will exceed 18 deaths per 100,000 and 0.01% chance that the cPIM will exceed 24 deaths per 100,000,

### Emergency department visits in a network of hospitals

Daily numbers of emergency department visits were obtained between the 1^st^ July 2004 to the 30^th^ October 2014 from the cyber-urgence network of “Assistance Publique/Hôpitaux de Paris” [10]. Thirty-seven hospitals participated to the network and reported, on a daily basis, the total number of visits in the emergency departments. Because of expected variations of the number of visits according to the day of the week, we considered weekly increments of emergency visits (iEV), that is, the difference between the number of visits on the same weekday between two consecutive weeks (S1 Fig).

We identified iEVM as the monthly maximal increase of the number of emergency department visits between the same weekdays from two consecutive weeks (n = 124). The empirical distribution of iEVM had a mean of 375 (minimum 82; maximum 1133) visits. The estimated location parameter was 305, 95%CI (282; 327), the scale parameter was 116, 95%CI (100; 133), and the shape parameter was 0.02, 95%CI (-0.09; 0.14). The fit was good (KS test P-value = 0.37) except for the observed highest maximum (S2 Fig). The return level plot showed here again a linear aspect due to the zero scale parameter (S2C Fig). We estimated a return level of 803, 95%CI (675; 931) visits and 984, 95%CI (762; 1206) visits at 5 and 20 years (60 and 240 months), respectively. The monthly risk of an increase greater than 1000 in the number of emergency department visits between the same weekday from two consecutive weeks is estimated to be 0.37%; it is 0.08% for an increase greater than 1200 visits.

## Discussion

Using simple illustrative examples, we showed the applicability of EVT to epidemiologic data. A GEV distribution was fitted to block maxima and was used to calculate estimates of return levels and of risks of exceeding a defined threshold value over given time periods.

In this work, we assumed the stationarity of the underlying working time series. This was likely the case for the two applications presented: means and standard deviations calculated over moving windows of different lengths did not vary over the study periods and the autocorrelations coefficients for both time series decreased rapidly towards the null (results not shown). Moreover, the model fits were good except for one outlier value of weekly increment of emergency department visits in summer 2014.

Methods for dealing with non-stationary distributions of maxima have been suggested in EVT. For other applications, it might be useful to consider a cyclical GEV model, that is a GEV model with time-varying location and scale parameters [2, 3, 5]. Yet, this method requires the estimation of at least two more parameters than the model presented in this paper: this might produce large confidence intervals due to the small numbers of observations available. To improve forecasting with relatively few annual observations, one might leverage other available information that could represent covariates associated with the outcome [3] (e.g. temperature, humidity levels, vaccine coverage… for P&I mortality or epidemics, heat waves, disasters, strikes or holidays, …for emergency department visits).

While such refinements might improve the accuracy of extreme value estimates, they are beyond the scope of this study as the choice of the specific approach would depend on the intended use of the forecasts.

Return level estimates should be helpful in planning resource needs, much like the statistical rationale for building dikes in the Netherlands. In our illustrative application on emergency department visits, EVT can be useful to estimate the surge capacity of the institution. For example, one could recommend sizing complementary health care resources (beds, staff) on a value that might be exceeded once in the next ten years–in our case an increase of 893 visits in the emergency rooms. Taking the example of seasonal influenza epidemics, if one assumes that antivirals, vaccines or face-masks stockpiles should be amassed, they can easily be dimensioned using estimates of EVT analysis based on an annual risk of exceeding an *a priori* defined threshold of cumulative influenza incidence. Other examples of potential applications include anticipating the impact of extreme environmental exposures such as heat waves, pollutants, radiations…

Nevertheless, a limitation of the method for public health planning is that it can’t be used to predict extreme events when these events differ by nature from those observed–as no distribution of related maxima will be observed and consequently, fitted. This means, for example, that EVT won’t help to anticipate what the impact could be of a nuclear disaster on emergency visits or to predict the mortality burden of an avian H5N1 influenza pandemic. For these types of extreme events, other methods such as risk analysis or modeling should be used. However, when data are available, we believe that extreme value theory offers a statistical rationale for public health planning of extreme events, and could be applied to a various range of topics in epidemiology.

## Supporting Information

S1 Fig Weekly increments of emergency visits (2004–2010) in Paris. iEV correspond to black symbols and iEVM, the monthly maxima, to the red symbols.
(PDF)

S2 Fig Fit of the Generalized Extreme Value distribution to the monthly maxima of iEV. a-Empirical (bars) and fitted (curve) distributions for the monthly maxima of iEV. b-Quantile-Quantile (QQ) plots for the monthly maxima of iEV. c- Return plots for the monthly maxima of iEV.
(PDF)

S1 File Pneumonia and Influenza data. Contains Flu season, date and 8-week sum of the age-standardized P&I death rates.
(CSV)

S2 File Emergency visits data. Contains date and number of emergency visits.
(CSV)
