# Supplementary figures and images for: Applications of Extreme Value Theory in Public Health
Source: PLoS One. 2016 Jul 15;11(7):e0159312. doi: 10.1371/journal.pone.0159312 (PMC4946775; doi:10.1371/journal.pone.0159312)

Weekly Increments of Entries in Emergency Rooms

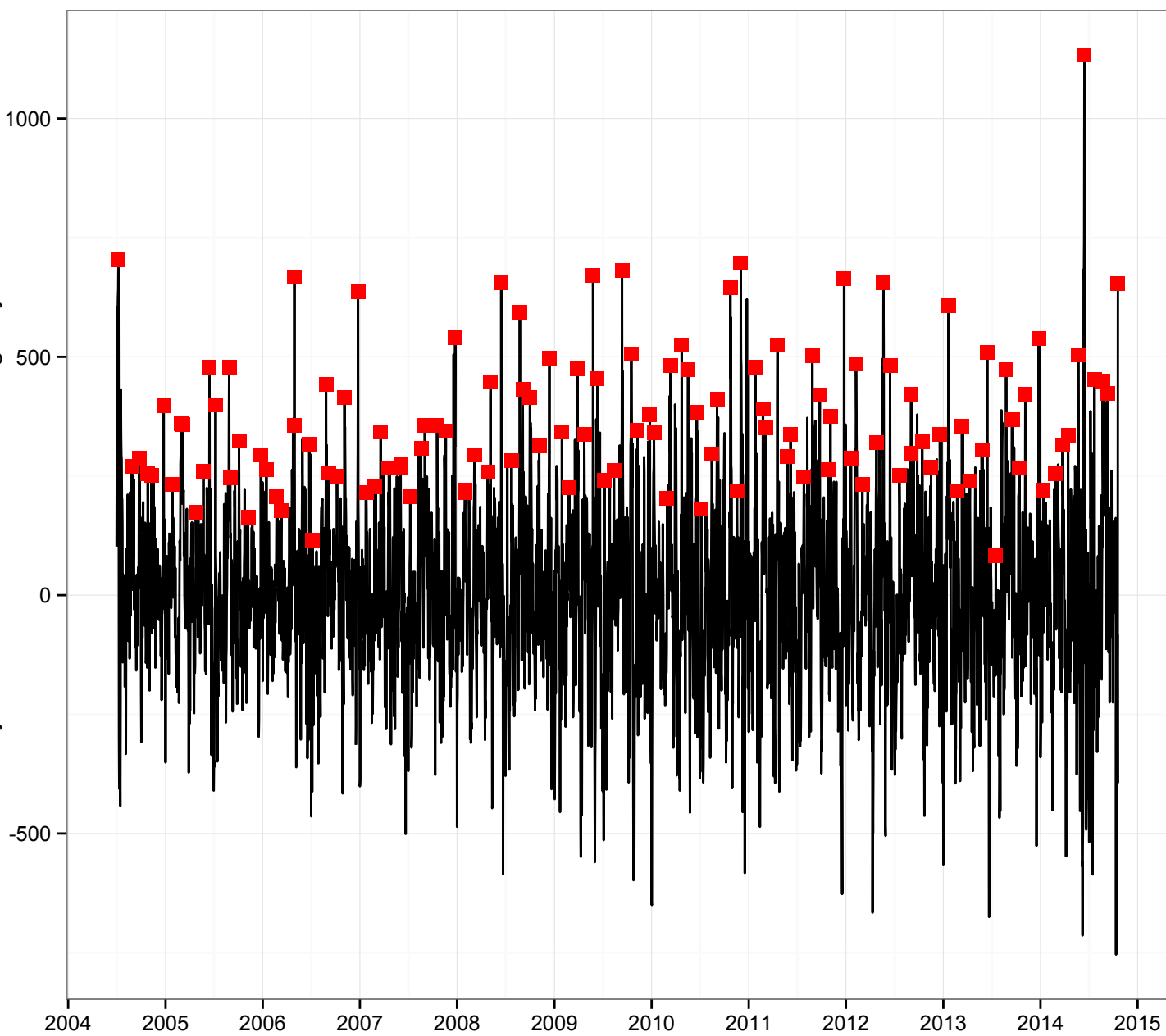

Maximum of Increments

Supplement: S1 Fig — iEV correspond to black symbols and iEVM, the monthly maxima, to the red symbols. (PDF) [file pone.0159312.s001.pdf]

a-Observed and Fitted distributions

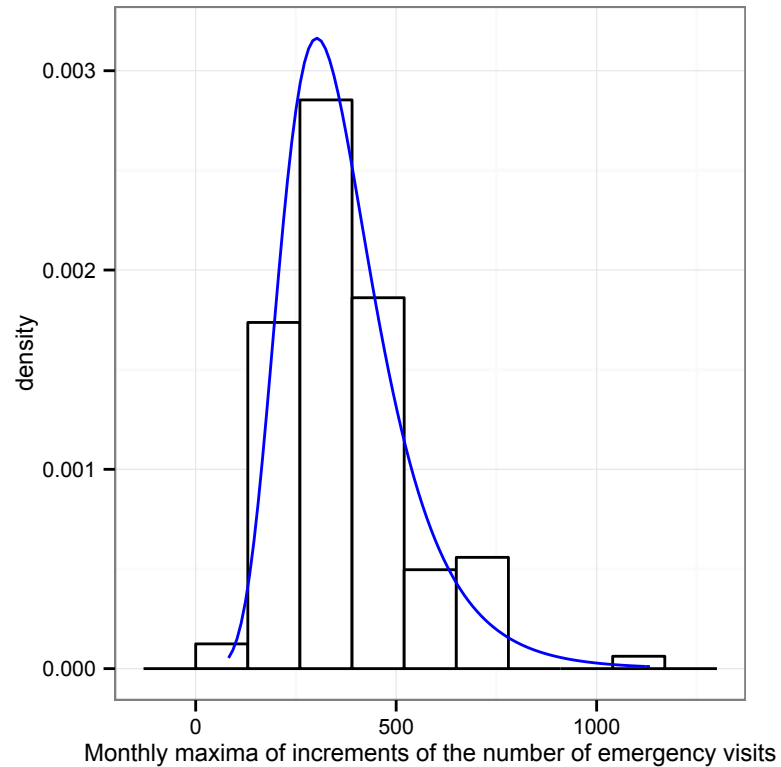

b-Quantile-Quantile plot

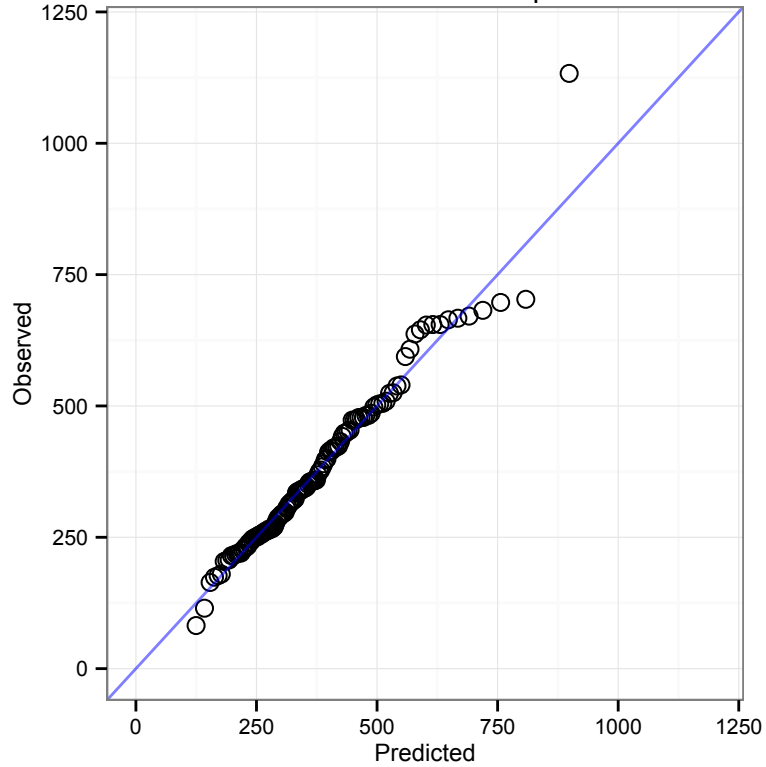

c-Return Level Plot

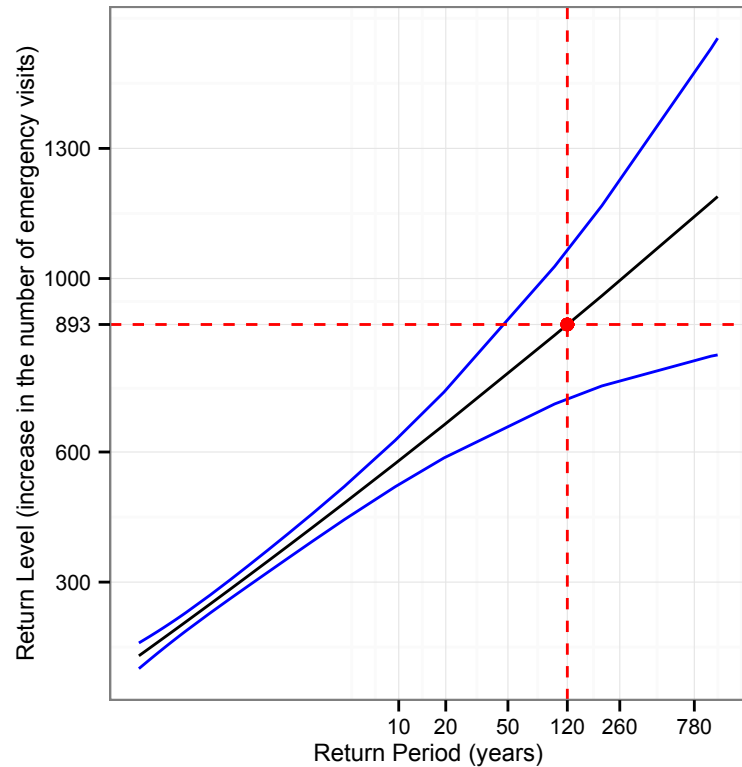

Supplement: S2 Fig — a-Empirical (bars) and fitted (curve) distributions for the monthly maxima of iEV. b-Quantile-Quantile (QQ) plots for the monthly maxima of iEV. c- Return plots for the monthly maxima of iEV. (PDF) [file pone.0159312.s002.pdf]
